# Serum sulfatide level is associated with severe systemic vasculitis with kidney involvement

**DOI:** 10.3389/fimmu.2023.1271741

**Published:** 2023-12-04

**Authors:** Daiki Aomura, Makoto Harada, Takero Nakajima, Takayuki Nimura, Kosuke Yamaka, Yosuke Yamada, Koji Hashimoto, Naoki Tanaka, Yuji Kamijo

**Affiliations:** ^1^Department of Nephrology, Shinshu University School of Medicine, Matsumoto, Japan; ^2^Department of Metabolic Regulation, Shinshu University School of Medicine, Matsumoto, Japan; ^3^Center for Medical Education and Training, Shinshu University School of Medicine, Matsumoto, Japan; ^4^Department of Global Medical Research Promotion, Shinshu University Graduate School of Medicine, Matsumoto, Japan; ^5^International Relations Office, Shinshu University School of Medicine, Matsumoto, Japan; ^6^Research Center for Social Systems, Shinshu University, Matsumoto, Japan

**Keywords:** kidney injury, rapidly progressive glomerulonephritis, sulfatides, sphingolipids, vasculitis

## Abstract

Sulfatides are a type of sulfated glycosphingolipid that are secreted with lipoproteins into the serum. These molecules are involved in the inflammatory pathway of vessels in addition to coagulation and platelet aggregation. Previous studies have proposed that sulfatides play a pivotal role in regulating inflammation-related disorders. Systemic vasculitis (SV) diseases are generally caused by autoimmune diseases and often involve kidney vasculitis, which may lead to rapidly progressive kidney dysfunction and end-stage kidney disease. Our earlier pilot study revealed that the level of serum sulfatides (SSs) was significantly decreased in patients with anti-neutrophil cytoplasmic antibody-associated vasculitis (AAV), a representative disease-causing SV with kidney involvement (SVKI), especially in patients exhibiting active crescentic findings on kidney biopsy. To further explore the clinical significance of an association between SS and SVKI, we analyzed and compared the SS level of patients with various SVKI diseases in this retrospective cohort study. Among patients admitted to our hospital between 2008 and 2021, we ultimately enrolled 26 patients with IgA vasculitis (IgAV), 62 patients with AAV, and 10 patients with anti-glomerular basement membrane disease (GBM) as examples of SVKI diseases, as well as 50 patients with IgA nephropathy (IgAN) and 23 donors for living kidney transplantation as controls. The mean ± standard deviation SS level in the donor, IgAN, IgAV, AAV, and GBM groups was 8.26 ± 1.72, 8.01 ± 2.21, 6.01 ± 1.73, 5.37 ± 1.97, and 2.73 ± 0.99 nmol/mL, respectively. Analysis of patients in the SVKI disease group showed that those with the crescentic class kidney biopsy finding exhibited a significantly lower SS level than did those with other class biopsy features. Additionally, the SS level had a higher detection ability for SVKI patients with crescentic class kidney biopsy findings (area under the receiver operating characteristic curve 0.90, 95% confidence interval 0.82–0.99) than did several other predictor candidates. Our results indicate that the SS level is decreased in more severe SVKI diseases and may be associated with active glomerular lesions in SVKI kidney biopsy samples.

## 1 Introduction

As a type of glycosphingolipid, 3-O-sulfogalactosylceramides (sulfatides) are abundantly found in the brain, kidney, and digestive tract as well as on platelet surfaces (1, 2). These lipids are also synthesized in the liver and secreted into the serum with lipoproteins (3, 4). Serum sulfatides (SSs) play a pivotal role in regulating inflammation and thrombogenesis pathways in blood vessels (1). Accordingly, several studies have associated the SS level with the severity of inflammation-related and cardiovascular diseases (5–9).

Immunological abnormalities can cause severe inflammation in vessels, leading to the development of systemic vasculitis (SV). SV diseases are generally classified according to the size of the targeted vessels. Those especially affecting small vessels, such as IgA vasculitis (IgAV), anti-neutrophil cytoplasmic antibody (ANCA)-associated vasculitis (AAV), and anti-glomerular basement membrane disease (GBM), often severely injure glomerular capillaries, causing rapidly progressive kidney dysfunction and the development of end-stage kidney disease (10–12). Given that vasculitis activates platelets and inflammation-related molecules (13), SSs are thought to be related to SV diseases with kidney involvement (SVKI). Our earlier pilot study revealed that the SS level in patients with AAV was significantly lower than that in healthy controls, and that a lower SS level was associated with active pathological findings on kidney biopsy (2). Those results suggested a significant contribution of SSs in the regulation of SVKI as well as a potential clinical utility of the SS level measurement. However, the study contained a limited sample size and only included patients with AAV; additional research, especially including SVKI diseases other than AAV, is required for further discussion on the relationship of SSs with SVKI.

The present study analyzed and compared the SS level of patients with and without SVKI diseases along with that in healthy controls. As an additional analysis, we evaluated associations of the SS level with both clinical and kidney biopsy findings to explore the clinical significance of the SS level measurement in the management of SVKI diseases.

## 2 Methods

### 2.1 Study design

This was a retrospective, single-center, observational study conducted at Shinshu University Hospital, Japan. The inclusion criteria of this study were as follows: 1) patients admitted to the Department of Nephrology at our hospital between 1 January 2008 and 31 December 2021; 2) patients firstly diagnosed as having IgA nephropathy (IgAN), IgAV, AAV, or GBM or who were living donors for kidney transplantation (donors); and 3) at least 20 years old at admission. The IgAV, AAV, and GBM groups were defined as the SVKI disease group, while the donor and IgAN groups were considered the non-SVKI disease group. All donor candidates for living kidney transplantation received medical screening tests at the hospital within 1 year before the operation, and those who passed the tests and completed the procedure by 31 December 2022 were included in the study. We randomly selected 50 IgAN patients from among 189 candidates for the analysis. Ten donors and 35 patients with AAV who were admitted between 1 January 2013 and 31 December 2019 and evaluated in our previous study (2) were also included in the present investigation.

### 2.2 Collection of patient data

Patient data were collected from hospital electrical medical records for age, body mass index (BMI), coexistence of hypertension and diabetes mellitus, vital signs, physical findings, and laboratory data at admission. Mean blood pressure (BP) was calculated as follows: mean BP = diastolic BP + 1/3 (systolic BP – diastolic BP). Hypertension was defined as antihypertensive drug prescription and/or a history of hypertension as described in the medical records. Diabetes mellitus was defined as elevated hemoglobin A1c (>6.5%), insulin or hypoglycemic agent prescription, and/or history of diabetes mellitus listed in the medical records. Birmingham Vasculitis Score 3 (BVAS), a scoring system for SV disease activity, was evaluated in SVKI disease group patients referring to medical records (14), with those scoring only in the renal section categorized as having renal-limited vasculitis (RLV) (15). Interstitial lung lesions were defined as bilateral interstitial lesions in computed tomography images. We also calculated Five-Factor Score (FFS), another SV severity scoring system, in the SVKI disease group based on age (>65 years old), cardiac and gastrointestinal involvement, renal insufficiency [serum creatinine (Cr) ≥1.6 mg/dL], and absence of ear-nose-throat manifestations as described in the medical records (16). Although BVAS and FFS were basically established for the assessment of AAV, we also applied them for evaluating IgAV and GBM to uniformly determine the activity of multiple SVKI diseases. Estimated glomerular filtration rate (eGFR) was calculated using a previously reported formula (17), and patients were classified according to Kidney Disease Improving Global Outcomes (KDIGO) guidelines: eGFR ≥90 mL/min was defined as G1, 60–89 mL/min as G2, 45–59 mL/min as G3a, 30–44 mL/min as G3b, 15–29 mL/min as G4, and <15 mL/min or hemodialysis treatment as G5 (18). Classification of total urine protein level was also conducted: patients with total urine protein level <0.15 g/gCr were defined as A1, 0.15–0.49 g/gCr as A2, and ≥0.5 g/gCr as A3. ANCA titer has been assessed at our institution as myeloperoxidase (MPO-) or proteinase 3 (PR3-) ANCA titer using a chemiluminescence enzyme immunoassay since 1 January 2013 and was determined as perinuclear (P-) or cytoplasmic ANCA until then. Information for each patient on treatment, death, and renal death was collected until 31 December 2022. Renal death was defined as the requirement for maintenance renal replacement therapy or kidney transplantation.

### 2.3 Definition and classification of pathological findings on kidney biopsy

Except for the donor group, pathological findings on kidney biopsy during hospitalization were collected referring to the biopsy report completed by renal pathologists. Information on light-microscopic findings, including the number of total glomeruli, normal glomeruli, glomeruli with the features of cellular crescent, fibrocellular crescent, fibrous crescent, mesangial hypercellularity, endocapillary hypercellularity, segmental sclerosis, and global sclerosis, the presence of arterial fibrinoid necrosis, and the percentage of interstitial fibrosis and tubular atrophy (IFTA) area, was extracted. A normal glomerulus was judged as one without crescents, mesangial hypercellularity, endocapillary hypercellularity, segmental sclerosis, global sclerosis, and glomerular basement membrane changes including membranous changes, rupture, duplication, and fibrinoid necrosis. In the IgAN group, we classified patients according to their pathological scores in the Oxford classification, which included cellular or fibrocellular crescent score (C score), mesangial hypercellularity score (M score), endocapillary hypercellularity score (E score), segmental glomerulosclerosis score (S score), and tubular atrophy/interstitial fibrosis score (T score) (19). In the SVKI disease group, we evaluated kidney biopsy findings according to the Berden classification system, a previously suggested classification method for the assessment of glomerulonephritis with SVKI diseases (20, 21). Based on the algorithm of this method, we divided kidney findings into four groups, as follows: biopsies containing >50% glomeruli with global sclerosis were categorized as sclerotic class; biopsies containing >50% normal glomeruli as focal class; biopsies containing >50% glomeruli with cellular and fibrocellular crescent findings as crescentic class; and biopsies without the features in the other categories as mixed class. Although the Berden classification has been generally used for the assessment of AAV and GBM, we also applied it for IgAV to uniformly compare the severity of crescent formation on kidney biopsy among multiple SVKI diseases. Additionally, we classified the patients in those groups depending on the presence of arterial fibrinoid necrosis and the percentage of IFTA (≥25% or not). In the IgAV group, classification by the International Study of Kidney Disease in Children (ISKDC) grade was also conducted (grade I, minimal alterations; grade II, mesangial proliferation, grade III, (a) focal or (b) diffuse proliferation or sclerosis with <50% crescents; grade IV, (a) focal or (b) diffuse proliferation or sclerosis with 50%–75% crescents; grade V, (a) focal or (b) diffuse proliferation or sclerosis with >75% crescents; and grade VI, membranoproliferative glomerulonephritis) (22).

### 2.4 Measurement of the SS level

Frozen serum samples obtained from patients at admission were used for the measurement of the SS level by a matrix-assisted laser desorption ionization time-of-flight mass spectrometry (MALDI-TOF MS) system. The analysis of lipids remains challenging due to their diversity and complexity, with numerous analytical methods available, including gas/liquid chromatography, nuclear magnetic resonance, enzyme-linked immunosorbent assays, and MS (23). Considering this was a translational study to newly establish an SVKI activity marker, the SS level was measured with the established MS-based method owing to its high performance for sensitivity, specificity, and accuracy (23). All measurements were conducted as described in our previous study (24) with a slight modification. Briefly, 50 μL of serum from subjects was mixed with 18 volumes of n-hexane/isopropanol solution (3:2, v/v) (24) for total lipid extraction. To quantify sulfatides, pooled normal human serum (#12181201, lot#BJ10633A, Cosmobio, Tokyo, Japan) was used as a standard, and total lipids were extracted in the same manner as above. We had determined the sulfatide concentration of the pooled human serum prior to this study. Afterward, lipid extracts from the samples and standard serum were treated with methanolic sodium hydroxide while being heated to convert sulfatides to their corresponding lysosulfatides (LSs; sulfatides without fatty acids). The LS samples were purified using Mono-tip C18 cartridges (GL Sciences, Tokyo, Japan), and equal amounts of an N-acetylated LS-sphinganine (LS-d18:0-NAc) calibrator were added to each sample. After drying, the LS samples were dissolved in a 9-aminoacridine matrix solution (5 mg/mL in 80% methanol; #92817, Merck, Darmstadt, Germany), and 1 µL of each sample was spotted on a MALDI-TOF MS plate. MALDI-TOF MS analysis of LS molecules was performed using a TOF/TOF 5800 system (AB Sciex, Framingham, MA, USA) in negative ion reflector mode with 2-point external calibration using the calibrator LS-d18:0-NAc [(M–H)^-^ 584.310] and LS-(4E)-sphingenine (d18:1) [(M–H)^-^ 540.284] peaks. As in our previous study, we detected seven LS species in normal human serum. Among those, LS-sphingadienine (d18:2), d18:1, and phytosphingosine (t18:0) were the major constituents, comprising more than 80% of the LS species (24). Thus, we focused on those three species in this study. The concentrations of LS-d18:2, d18:1, and t18:0 in each sample were calculated using the data of the standard serum, and their sum was defined as SS concentration. For each serum sample, LS samples were prepared in duplicate or triplicate, and at least two spots were analyzed for each replicate in MALDI-TOF MS analysis.

### 2.5 Statistical analysis

All continuous variables were evaluated by the Shapiro–Wilk test for normality, and those exhibiting normal distribution were presented as the mean and standard deviation (SD). Those exhibiting a non-normal distribution were presented as the median and interquartile range (IQR). Comparisons of continuous variables were conducted using Student’s *t*-test or the Mann–Whitney U test according to whether they exhibited a normal or non-normal distribution, respectively. For comparing continuous variables in more than two groups, we assessed the differences between all sets of two groups using Student’s *t*-test or the Mann–Whitney U test. In the analysis of patient characteristics, however, we evaluated the difference among all disease groups by one-way analysis of variance. Categorial variables were presented as the number and percentage, and the chi-square test or Fisher’s exact probability test was used for comparisons. While comparing the SS level between disease groups, we also conducted multiple regression analysis between every two groups, calculating the partial regression coefficient of the difference in the disease group for the SS level after adjusting for age and sex. To confirm the factors related to the SS level, we evaluated the correlation coefficient between the SS level and various basic patient background parameters (age and BMI), hepatic metabolism [serum albumin, aspartate aminotransferase (AST), alanine aminotransferase (ALT), gamma-glutamyl transpeptidase (γ-GTP), and total bilirubin (T-bil) level], cholesterol metabolism [serum total cholesterol (TC), high-density lipoprotein cholesterol (HDL-C), low-density lipoprotein cholesterol (LDL-C), and triglyceride (TG) level], kidney injury (eGFR and urine protein level), inflammation [BVAS and serum C-reactive protein (CRP) level], thrombogenesis (platelet count and D-dimer level), and kidney biopsy findings (percentage of normal glomeruli, glomeruli with the cellular crescent finding, glomeruli with the cellular or fibrocellular crescent finding, glomeruli with all-type crescent findings, and glomeruli with global sclerosis, as well as IFTA). Pearson or Spearman correlation analysis was employed depending on normal or non-normal variable distribution, respectively. Those analyses were conducted on patients in each disease group, all patients in the non-SVKI disease group, and all patients in the SVKI disease group. We additionally conducted multiple regression analysis of the SVKI disease patients to search for associations of the SS level with parameters related to crescent findings after adjusting for age, sex, renal dysfunction (eGFR), hepatic injury (serum ALT level), and lipid metabolism (serum TC level). As there were missing values for T-bil, TC, HDL-C, LDL-C, and D-dimer level, we replaced those with substituted plausible values by the univariate imputation method using the mice 3.15 package on R, also conducting the correlation coefficient analysis with the imputed dataset as a sensitivity analysis.

The SS level was compared between groups classified according to clinical findings, outcome development, and kidney biopsy findings. In the assessment of patients in the SVKI disease group, the SS level of patients with crescentic class in the Berden classification was compared with that of patients with each non-crescentic class (focal, mixed, and sclerotic class) and the level of all patients with any non-crescentic class. Additionally, the detection ability of the SS level in patients with crescentic class was evaluated by receiver operating characteristic (ROC) curve analysis for calculation of the area under the ROC curve (AUC). The AUC value for the SS level was compared with those of other possible predictors, including CRP, eGFR, BVAS, and FFS, using DeLong’s test. The detection ability of combined formulae calculated with the SS level and each predictor was also evaluated. Each combined formula was built using a logistic regression model that automatically showed the highest AUC values. The AUC values of the combined formulae were compared with those of each predictor alone. Such testing was conducted on patients in each SVKI disease group as well as on all patients in the SVKI disease group.

As a *post-hoc* analysis, we evaluated the association of sulfatide component ratios (i.e., the proportion of serum d18:2, d18:1, and t18:0 levels per total SS level) with crescentic findings on kidney biopsy. The correlation coefficients between the ratios and the percentage of glomeruli with crescent findings were calculated. Furthermore, sulfatide component ratios were compared among groups classified according to the C score in the Oxford classification in IgAN patients as well as among those classified according to the Berden classification in patients of the IgAV, AAV, GBM, and SVKI disease groups.

Statistical significance was set at *p* < 0.05, and all analyses were performed using R software (version 4.2.2, R Foundation for Statistical Computing, Vienna, Austria).

### 2.6 Ethical approval

This study was approved by the institutional review board of the ethics committee of Shinshu University School of Medicine (approval number: 5419) and was conducted in accordance with the principles of the Declaration of Helsinki. The requirement for written informed consent was waived because of the retrospective nature of the study. The data used in this investigation are available from the corresponding author upon reasonable request.

## 3 Results

### 3.1 Patient characteristics

**Figure 1:**
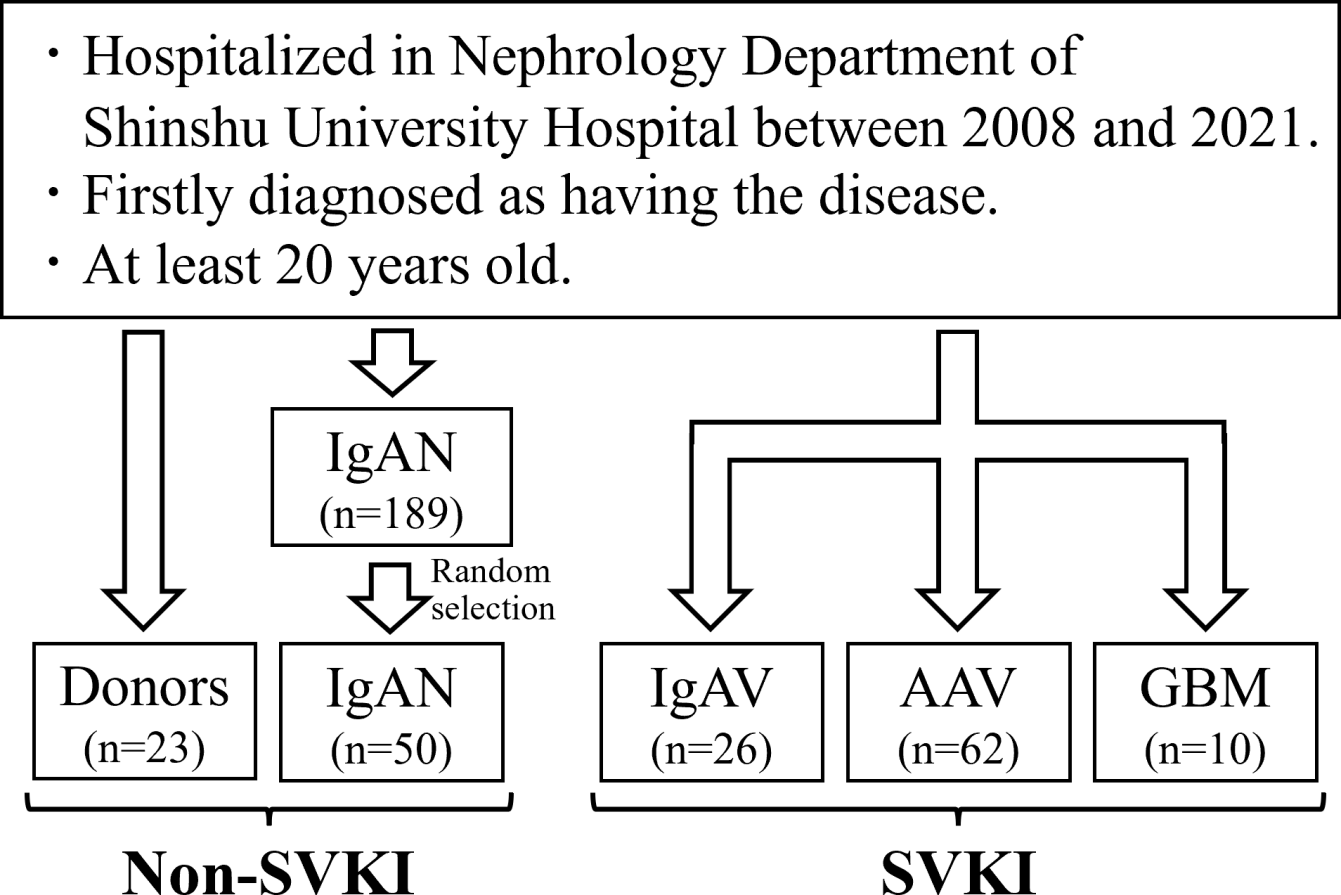
Study flowchart. The total number of eligible patients was 171. AAV, anti-neutrophil cytoplasmic antibody-associated vasculitis; GBM, anti-glomerular basement membrane disease; IgAN, IgA nephropathy; IgAV, IgA vasculitis; SVKI, systemic vasculitis with kidney involvement.

The flowchart of this study is shown in **Figure 1**. We analyzed 171 patients comprising 23 donors, 50 patients with IgAN, 26 patients with IgAV, 62 patients with AAV, and 10 patients with GBM. The cohort’s characteristics are summarized in **Table 1**. Mean ± SD age was 56.8 ± 8.4 years, 41.0 ± 11.9 years, 52.4 ± 19.9 years, 73.9 ± 10.6 years, and 63.2 ± 18.3 years and male proportion was 43.5%, 58.0%, 53.8%, 54.8%, and 20.0% in the donor, IgAN, IgAV, AAV, and GBM groups, respectively. The SVKI disease groups generally exhibited lower albumin, eGFR, T-bil, cholesterols, and hemoglobin levels, as well as higher BP, Cr, CRP, white blood cell, platelet, fibrinogen, D-dimer, and urine protein levels than did the non-SVKI disease groups. Kidney biopsy was performed on 50 (100%) patients in the IgAN group, 25 (93.2%) patients in the IgAV group, 45 (72.6%) patients in the AAV group, and 8 (80.0%) patients in the GBM group. No death or renal death was observed in the donor or IgAN groups but was witnessed in 1 (6.8%) and 0 patients in the IgAV group, 10 (16.1%) and 19 (30.6%) patients in the AAV group, and 1 (10.0%) and 7 (70.0%) patients in the GBM group.

**Table 1:** Patient characteristics.

	Donor (n=23)	IgAN (n=50)	IgAV (n=26)	AAV (n=62)	GBM (n=10)	*p*
Age (years)	56.8 ± 8.4	41.0 ± 11.9	52.4 ± 19.9	73.9 ± 10.6	63.2 ± 18.3	<0.001
Male (n)	10 (43.5)	29 (58.0)	14 (53.8)	34 (54.8)	2 (20.0)	0.22
BMI (kg/m^2^)	22.8 ± 3.1	23.6 ± 3.5	25.0 ± 4.0	22.1 ± 3.4	24.1 ± 5.5	0.01
Body temperature (°C)	36.5 ± 0.31	36.6 ± 0.37	36.7 ± 0.62	36.7 ± 0.50	36.9 ± 0.78	0.11
Mean blood pressure (mmHg)	87.3 ± 10.8	89.9 ± 12.1	91.5 ± 14.3	99.2 ± 15.6	102.4 ± 19.4	<0.001
Hypertension (n)	4 (17.4)	19 (38.0)	11 (42.3)	42 (67.7)	4 (40.0)	<0.001
Diabetes mellitus (n)	3 (13.0)	1 (2.0)	5 (19.2)	15 (24.2)	0 (0.0)	0.009
BVAS	N/A	N/A	11.0 [11.0, 13.8]	17.5 [14.0, 19.8]	16.5 [16.0, 18.8]	<0.001
FFS	N/A	N/A	1.0 [1.0, 1.0]	2.0 [1.0, 2.0]	2.0 [2.0, 2.0]	<0.001
Laboratory data						
Albumin (g/dL)	4.36 ± 0.31	3.96 ± 0.52	3.45 ± 0.81	2.88 ± 0.69	2.63 ± 0.49	<0.001
Cr (mg/dL)	0.72 [0.64, 0.81]	0.97 [0.80, 1.3]	0.88 [0.66, 1.09]	3.14 [1.83, 5.20]	5.98 [4.97, 7.21]	<0.001
eGFR (mL/min/1.73m^2^)	75.0 [69.5, 80.0]	63.5 [49.3, 84.0]	69.0 [58.8, 78.0]	15.0 [9.0, 24.8]	6.50 [5.3, 7.8]	<0.001
T-bil (mg/dL)	0.69 ± 0.20	0.65 ± 0.23	0.60 ± 0.31	0.51 ± 0.25	0.42 ± 0.18	0.005
AST (IU/L)	19.0 ± 6.7	19.6 ± 7.9	26.2 ± 45.2	30.6 ± 90.8	39.9 ± 44.8	0.48
ALT (IU/L)	21.6 ± 7.03	17.8 ± 11.4	22.6 ± 24.3	27.1 ± 35.6	30.4 ± 22.0	0.70
γ-GTP (IU/L)	31.3 ± 18.8	24.3 ± 15.2	46.5 ± 66.3	32.7 ± 37.2	70.6 ± 89.1	0.01
CRP (mg/dL)	0.03 [0.01, 0.08]	0.04 [0.02, 0.12]	0.49 [0.10, 1.61]	2.01 [0.34, 6.08]	6.26 [3.40, 14.16]	<0.001
TC (mg/dL)	213 ± 33	201 ± 40	211 ± 39	181 ± 38	164 ± 39	<0.001
HDL-C (mg/dL)	59.7 ± 14.8	57.0 ± 14.8	56.3 ± 13.9	42.1 ± 15.2	37.2 ± 10.6	<0.001
LDL-C (mg/dL)	121.5 ± 28.3	117.7 ± 31.0	128.0 ± 35.8	106.3 ± 27.8	92.1 ± 22.6	0.004
TG (mg/dL)	139 ± 64	143 ± 92	132 ± 58	135 ± 58	142 ± 92	0.97
White blood cell (/μL)	5517 ± 1469	6387 ± 2412	7836 ± 4136	9215 ± 5444	10074 ± 3317	<0.001
Hemoglobin (g/dL)	14.2 ± 1.0	13.9 ± 1.7	16.9 ± 2.0	9.95 ± 1.76	9.10 ± 1.82	0.001
Platelet (×10^4^/μL)	24.6 ± 4.9	25.2 ± 5.9	28.7 ± 8.5	28.5 ± 10.7	35.3 ± 13.2	0.005
Fibrinogen (mg/dL)	273 [249, 297]	285 [254, 330]	373 [293, 501]	425 [346, 545]	557 [422, 787]	<0.001
D-dimer (μg/mL)	0.50 [0.50, 0.60]	0.60 [0.50, 0.80]	1.30 [0.75, 3.65]	4.20 [2.10, 7.10]	5.60 [4.07, 11.05]	<0.001
Urine protein (g/gCr)	0.00 [0.00, 0.00]	0.65 [0.37, 1.32]	0.87 [0.33, 2.89]	1.64 [0.94, 3.33]	1.88 [1.38, 2.76]	<0.001
Treatment pattern						
Kidney biopsy (n)	N/A	50 (100.0)	25 (93.2)	45 (72.6)	8 (80.0)	<0.001
Methylprednisolone pulse (n)	0 (0.0)	37 (74.0)	17 (65.4)	45 (72.6)	9 (90.0)	<0.001
Azathioprine (n)	0 (0.0)	0 (0.0)	0 (0.0)	9 (14.5)	2 (20.0)	0.002
Cyclophosphamide (n)	0 (0.0)	0 (0.0)	3 (11.5)	9 (14.5)	5 (50.0)	<0.001
Rituximab (n)	0 (0.0)	0 (0.0)	0 (0.0)	9 (14.5)	4 (40.0)	<0.001
Hemodialysis (n)	0 (0.0)	0 (0.0)	0 (0.0)	23 (37.1)	8 (80.0)	<0.001
Plasma exchange (n)	0 (0.0)	0 (0.0)	0 (0.0)	7 (11.3)	10 (100)	<0.001

Continuous variables are presented as the mean ± SD or median [IQR], and categorial variables are presented as n (%). The *p*-value calculated by one-way analysis of variance for a difference among the disease groups is presented.AAV, anti-neutrophil cytoplasmic antibody-associated vasculitis; ALT, alanine aminotransferase; AST, aspartate aminotransferase; BMI, Body mass index; BVAS, Birmingham Vasculitis Activity Score; Cr, creatinine; CRP, C-reactive protein; eGFR, estimated glomerular filtration rate; FFS, five factor score; Foc, focal class; GBM, anti-glomerular basement membrane disease; γ-GTP, gamma-glutamyl transpeptidase; HDL-C, high density lipoprotein cholesterol; IgAN, IgA nephropathy; IgAV, IgA vasculitis; LDL-C, light density lipoprotein cholesterol; N/A, not assessed; T-bil, total bilirubin; TC; total cholesterol; TG, triglyceride.

### 3.2 The SS level of each disease group

**Figure 2:**
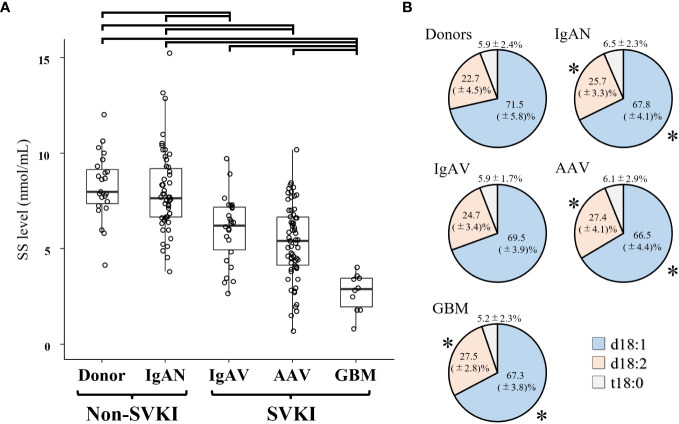
The SS level in each disease group. **(A)** The SS level and **(B)** proportion of component materials of SS in each disease group. Significant SS level differences between disease groups are indicated by horizontal brackets. * *p* < 0.05 vs. corresponding component in the donor group. AAV, anti-neutrophil cytoplasmic antibody-associated vasculitis; d18:1, sphingenine; d18:2, sphingadienine; GBM, anti-glomerular basement membrane disease; IgAN, IgA nephropathy; IgAV, IgA vasculitis; SS, serum sulfatides; SVKI, systemic vasculitis with kidney involvement; t18:0, phytosphingosine.

The mean ± SD SS level was 8.26 ± 1.72 nmol/mL in the donor group, 8.01 ± 2.21 nmol/mL in the IgAN group, 6.01 ± 1.73 nmol/mL in the IgAV group, 5.37 ± 1.97 nmol/mL in the AAV group, and 2.73 ± 0.99 nmol/mL in the GBM group (**Figure 2A**). The SS level in both the IgAV and AAV groups was significantly lower than that in the donor and IgAN groups, with the GBM group exhibiting a significantly lower SS level versus all other groups. The differences in the SS level between the disease groups remained significant even after adjusting for age and sex (**Supplementary Table S1**). Analysis of the sulfatide components in each disease group revealed a significantly lower d18:1 and higher d18:2 proportion in the IgAN, AAV, and GBM groups than that in the donor group (**Figure 2B**).

### 3.3 Correlations of the SS level with parameters related to clinical and kidney biopsy findings

Results on the correlations of the SS level with parameters concerning clinical and kidney biopsy findings in the non-SVKI and SVKI disease groups are shown in **Table 2**. The SS level was significantly correlated with TC, LDL-C (positively), ALT, and γ-GTP (negatively) levels in the non-SVKI disease group. In the SVKI disease group, the SS level was significantly and positively correlated with albumin, TC, HDL-C, LDL-C, and eGFR levels and negatively correlated with γ-GTP, CRP, and D-dimer levels. Furthermore, the SS level was significantly associated with the percentage of glomeruli displaying the cellular crescent finding and cellular or fibrocellular crescent finding on kidney biopsy in the SVKI disease group. Even after adjusting for age, sex, eGFR, and serum ALT and TC levels using multiple regression analysis, the SS level remained significantly associated with the percentage of active crescentic findings in the SVKI disease group (**Table 3**, **Supplementary Table S2**). The analysis results for each disease group are shown in **Supplementary Table S3**. Notably, the SS level was significantly and negatively correlated with the percentage of glomeruli having the finding of cellular crescents in patients with GBM [correlation coefficient r: -0.75, 95% confidence interval (CI), -0.95 to -0.10; *p* = 0.03]. The sensitivity analysis using an imputed dataset for missing values yielded similar results (**Supplementary Table S4**).

**Table 2:** Correlations of the SS level with parameters related to clinical and kidney biopsy findings.

	Non-SVKI disease group	*p*	SVKI disease group	*p*
Age (years)	0.02 (-0.21, 0.25)	0.86	-0.16 (-0.34, 0.04)	0.12
BMI (kg/m^2^)	0.03 (-0.20, 0.26)	0.82	-0.08 (-0.27, 0.12)	0.44
BVAS	N/A	N/A	-0.18 (-0.37, 0.02)	0.07
Laboratory data				
Albumin (g/dL)	-0.07 (-0.29, 0.17)	0.58	0.46 (0.28, 0.60)	<0.001
T-bil (mg/dL)	-0.11 (-0.35, 0.14)	0.37	0.04 (-0.17, 0.25)	0.70
AST (IU/L)	-0.12 (-0.34, 0.11)	0.31	-0.20 (-0.38, 0.00)	0.05
ALT (IU/L)	-0.23 (-0.43, -0.00)	0.05	-0.18 (-0.36, 0.02)	0.08
γ-GTP (IU/L)	-0.36 (-0.55, -0.15)	0.002	-0.28 (-0.45, -0.09)	0.01
TC (mg/dL)	0.36 (0.14, 0.55)	0.002	0.62 (0.48, 0.73)	<0.001
HDL-C (mg/dL)	0.20 (-0.06, 0.43)	0.13	0.53 (0.36, 0.66)	<0.001
LDL-C (mg/dL)	0.32 (0.09, 0.52)	0.008	0.49 (0.32, 0.63)	<0.001
TG (mg/dL)	-0.08 (-0.31, 0.16)	0.51	0.02 (-0.18, 0.22)	0.85
eGFR (mL/min/1.73m^2^)	0.01 (-0.22, 0.24)	0.93	0.23 (0.03, 0.41)	0.03
CRP (mg/dL)	0.00 (-0.23, 0.23)	0.98	-0.66 (-0.76, -0.53)	<0.001
Platelet (×10^4^/μL)	0.14 (-0.09, 0.36)	0.23	-0.06 (-0.26, 0.14)	0.56
D-dimer (μg/mL)	0.24 (-0.04, 0.48)	0.10	-0.43 (-0.59, -0.24)	<0.001
Urine protein (g/gCr)	-0.04 (-0.27, 0.19)	0.74	0.00 (-0.20, 0.20)	0.97
Pathological findings				
All-type crescents (%)	0.14 (-0.15, 0.40)	0.34	-0.20 (-0.40, 0.03)	0.08
Cellular crescents (%)	0.02 (-0.26, 0.30)	0.89	-0.28 (-0.47, -0.06)	0.01
Cellular or fibrocellular crescents (%)	0.10 (-0.18, 0.37)	0.49	-0.28 (-0.47, -0.06)	0.01
Global sclerosis (%)	0.28 (0.00, 0.52)	0.05	0.00 (-0.22, 0.22)	>0.99
Normal glomeruli (%)	-0.24 (-0.49, 0.04)	0.09	0.24 (0.02, 0.44)	0.03
IFTA (%)	0.05 (-0.23, 0.33)	0.71	-0.02 (-0.24, 0.20)	0.85

Correlation coefficient and 95% CI of the SS level with parameters concerning laboratory data and kidney biopsy findings.ALT, alanine aminotransferase; AST, aspartate aminotransferase; BMI, Body mass index; BVAS, Birmingham Vasculitis Activity Score; CRP, C-reactive protein; eGFR, estimated glomerular filtration rate; γ-GTP, gamma-glutamyl transpeptidase; HDL-C, high density lipoprotein cholesterol; IFTA, interstitial fibrosis and tubular atrophy; LDL-C, light density lipoprotein cholesterol; N/A, not assessed; SS, serum sulfatides; SVKI, systemic vasculitis with kidney involvement; T-bil, total bilirubin; TC; total cholesterol; TG, triglyceride.

**Table 3:** Correlation of the SS level with parameters related to crescentic kidney biopsy findings in the SVKI disease group in multivariate analysis.

	Crude model	*p*	Model 1		Mode 2	*p*
All-type crescents (%)	-0.04 (-0.06, -0.01)	0.006	-0.04 (-0.06, -0.01)	0.006	-0.03 (-0.06, -0.00)	0.04
Cellular crescents (%)	-0.03 (-0.05, -0.01)	<0.001	-0.03 (-0.05, -0.01)	<0.001	-0.03 (-0.05, -0.01)	0.004
Cellular or fibrocellular crescents (%)	-0.05 (-0.07, -0.02)	<0.001	-0.05 (-0.07, -0.02)	<0.001	-0.04 (-0.06, -0.01)	0.006

Partial regression coefficient of the SS level for crescentic kidney biopsy findings in the SVKI disease group adjusted for age and sex (model 1) or age, sex, eGFR, and serum ALT and TC levels (model 2) is described with 95% CI.

### 3.4 Associations of the SS level with clinical and kidney biopsy findings in each disease group

In IgAN patients, the SS level was similar among groups classified according to staging for GFR or urine protein level or based on kidney biopsy findings in the Oxford classification (**Supplementary Figure S1**). In the IgAV group, patients with accompanying abdominal pain showed a significantly lower SS level than did those without (**Supplementary Figure S2**). The SS level was comparable among groups classified according to other clinical findings [presence of arthritis, fever (>37.5°C), or purpura at admission, RLV type or not, and staging for GFR or urine protein level] and outcome development. In the analysis of crescentic findings on kidney biopsy, no patient was classified as exhibiting crescentic class in the Berden classification, and staging by the ISKDC classification was not associated with the SS level. The finding of arterial fibrinoid necrosis on kidney biopsy was not observed in any patient, and the SS level was similar between groups classified according to IFTA (over 25% or not).

Among AAV patients, 61 (98.4%) were positive for MPO- or P-ANCA and were diagnosed as having microscopic polyangiitis. One patient was positive for PR3-ANCA and was diagnosed as having granulomatosis with polyangiitis. The SS level was not associated with clinical findings (presence of lung findings, RLV type or not, or staging for GFR or urine protein level) or outcome development (**Supplementary Figure S3**). Patients with crescentic class in the Berden classification showed a significantly lower SS level than did those with mixed class or all patients with non-crescentic classes. The SS level was comparable between groups classified according to the presence of arterial fibrinoid necrosis or IFTA (over 25% or not).

In the GBM group, the SS level was not associated with clinical findings (presence of lung findings, RLV type or not, or staging for GFR or urine protein level) or outcome development (**Supplementary Figure S4**). In the analysis of kidney biopsy findings, patients with crescentic class in the Berden classification showed an SS level similar to those in patients classified as other classes. The SS level of groups classified according to the presence of arterial fibrinoid necrosis or IFTA (over 25% or not) was also comparable.

### 3.5 Detection ability of the SS level for SVKI patients with the crescentic class kidney biopsy finding

**Figure 3:**
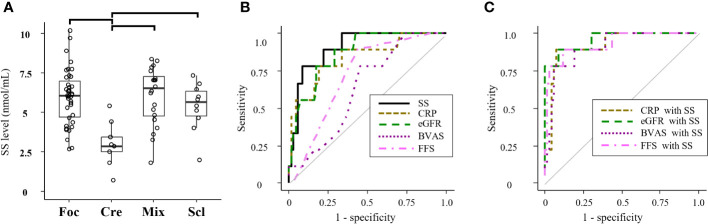
Associations of the SS level with kidney biopsy findings in all SVKI patients. **(A)** The SS level of groups classified by the Berden classification among all patients in the SVKI disease group. Significant differences are indicated by horizontal brackets. **(B)** ROC analysis of patients with the crescentic class kidney biopsy finding in the Berden classification by the SS level and other predictors and **(C)** by combination of the SS level with other predictors. BVAS, Birmingham Vasculitis Activity Score; Cre, crescentic class; CRP, C-reactive protein; eGFR, estimated glomerular filtration rate; FFS, five factor score; Foc, focal class; Mix, mixed class; Scl, sclerosis class; SS, serum sulfatides.

In the SVKI disease group, patients with the crescentic class kidney biopsy finding in the Berden classification displayed a significantly lower SS level than did those with focal, mixed, or sclerotic class findings (**Figure 3A**). ROC analyses of the SS level and other predictive markers in patients with the crescentic class finding are presented in **Figure 3B**. The AUC value for an SS level of 0.90 (95% CI 0.82–0.99) was similar to those for CRP and eGFR (**Table 4A**) and significantly higher than those for BVAS and FFS. Combined formulae with the SS level tended to improve the detection ability of CRP and significantly improved those of eGFR, BVAS, and FFS (**Figure 3C**, **Table 4B**).

**Table 4:** Detection ability of the SS level for SVKI patients with crescentic class kidney biopsy finding.

(A)	AUC value	*p*
SS	0.90 (0.82, 0.99)	Ref
CRP	0.84 (0.68, 0.99)	0.27
eGFR	0.87 (0.76, 0.97)	0.43
BVAS	0.64 (0.47, 0.80)	0.001
FFS	0.72 (0.60, 0.84)	<0.001
(B)	AUC value	*p*
CRP with SS	0.92 (0.84, 1.00)	0.08
eGFR with SS	0.96 (0.89, 1.00)	0.01
BVAS with SS	0.91 (0.82, 1.00)	<0.001
FFS with SS	0.93 (0.83, 1.00)	<0.001

AUC values with 95% CI for patients with the crescentic class kidney biopsy finding in the Berden classification in the SVKI disease group. (A) AUC values for possible predictors were compared with that for the SS level, and (B) the values of combined formulae for the SS level and other predictors were compared with the value for each predictor alone.AUC, area under the receiver operating characteristic curve; BVAS, Birmingham Vasculitis Activity Score; Cr, creatinine; CRP, C-reactive protein; eGFR, estimated glomerular filtration rate; FFS, five factor score; SS, serum sulfatides.

In the AAV group, the AUC value for the SS level was 0.86 (95% CI 0.80–1.00), which tended to be higher than those for CRP, eGFR, and BVAS and significantly higher than that for FFS (**Supplementary Figure S5A**, **Supplementary Table S5A**). Combined formulae with the SS level significantly improved the AUC value for eGFR and FFS (**Supplementary Figure S5B**, **Supplementary Table S5B**). The ROC results for the SS level and other predictors in the GBM group are displayed in **Supplementary Figure S5C**. The AUC value for the SS level was 0.67 (95% CI 0.01–1.00), which was similar to those for other predictors (**Supplementary Table S5A**). The AUC value by combining the SS level and FFS was significantly higher than that for FFS only (**Supplementary Figure S5D**, **Supplementary Table S5B**).

### 3.6 Associations of the sulfatide component ratio with crescentic findings on kidney biopsy

The proportions of d18:1 and d18:2 per total SS level were significantly correlated with the percentage of glomeruli with all-type crescents, cellular crescents, and cellular or fibrocellular crescents in the IgAN group (**Supplementary Table S6A**). Additionally, patients scoring C1 in the Oxford classification exhibited a significantly higher proportion of d18:2 than did those scoring C0 (**Supplementary Figure S6A**). In the SVKI disease group, the proportions of d18:1 and d18:2 were significantly correlated with the percentage of glomeruli with all-type crescents, but not of those with cellular crescents or cellular or fibrocellular crescents (**Supplementary Table S6B**). In the IgAV, AAV, and GBM groups, no sulfatide component ratios correlated with any parameters concerning crescentic findings (**Supplementary Tables S6C–S6E**). The crescentic class kidney biopsy finding in the Berden classification was not associated with the sulfatide component ratio in the SVKI disease, IgAV, AAV, or GBM groups (**Supplementary Figures S6B–S6E**).

## 4 Discussion

The present study revealed the SS level to be lower in patients with SVKI diseases than in those with non-SVKI diseases. Generally considered the most severe SVKI disease, GBM exhibited the lowest SS level compared with all other diseases. Our results support a close association of the SS level with SVKI severity and confirm a pathophysiological connection of sulfatides with vasculitis.

Although the precise mechanism of a connection between the SS level and vasculitis is uncertain, a possible hypothesis was proposed in our earlier study (2). Sulfatides are involved in the inflammatory pathway through interactions with L-selectin on leukocytes as well as with P-selectin on platelets and vascular endothelium (1). Vasculitis is known to cause the overexpression of selectins during severe inflammation (13). Under this premise, vasculitis is considered to consume SS in reactions with selectins, thus decreasing the SS level in patients with severe SVKI. As another hypothesis, SVKI might indirectly influence the SS level through the suppression of hepatic sulfatide synthesis. SSs are mainly produced in the liver, and several animal studies have described that kidney dysfunction and a subsequent increase in serum oxidative stress (OS) suppress the hepatic gene expression related to sulfatide synthesis (3, 25). Wang et al. (26) found that the SS level in patients with end-stage kidney disease was restored after kidney transplantation and a decrease in serum OS. Although serum OS was not measured in our study, SVKI might have decreased the SS level through kidney dysfunction, increased serum OS level, and suppressed sulfatide synthesis in the liver. In addition, sulfatides are known to be present within lipoproteins in the serum (3, 4), and serum lipoprotein levels were lower in the SVKI disease group, probably due to a poor nutrition state and impaired hepatic function caused by SVKI and severe systemic illness. The SS level and serum lipoprotein levels were generally well correlated in all of the disease groups. Considering this fact, the decreased SS level in the SVKI disease group might have reflected lower serum lipoprotein levels. Although the precise mechanism of the decreased SS level in SVKI diseases remains unclear, our results suggest that a lower SS level comprehensively reflects the severity of SVKI.

This study also examined for associations of the SS level with kidney biopsy results, focusing especially on the crescentic finding, a major indicator of SVKI activity. In the analysis of all patients in the SVKI disease group, the SS level was significantly lower in those whose kidney biopsy findings were classified as crescentic class in the Berden classification. Furthermore, we uncovered a sufficiently high detection ability of the SS level in patients with this finding. Such individuals reportedly suffer from poor survival and frequently require intensive treatment (20, 27). Our results implied that a lower SS level was associated with active crescentic findings in kidney biopsy samples of SVKI diseases. Thus, measuring the SS level could be helpful for the assessment of SVKI severity and disease management.

Different from the results of the SVKI disease groups, the SS level was not associated with crescentic findings on kidney biopsy in IgAN patients. Given that the percentage of glomeruli with cellular or fibrocellular crescents in the IgAN group was much lower than that in the SVKI disease groups (average percentage: 3.3%, 10.7%, 24.3%, and 58.7% in the IgAN, IgAV, AAV, and GBM groups, respectively), the relatively mild renal vasculitis observed in IgAN patients presumably did not influence the SS level. In addition, patients with IgAN generally do not experience vasculitis apart from in the kidney, and none in this study exhibited findings indicating SV, such as fever, abdominal pain, and high serum CRP level. Considering the hypothesis that SV and a subsequent severely ill condition could decrease the SS level, the lack of SV among IgAN patients might explain their relatively higher SS level and nonsignificant association of the SS level with pathological and clinical parameters.

The SS level was not related to kidney biopsy findings in the IgAV group according to the Berden or ISKDC classification. However, considering the fact that no patient was classified as having crescentic class in the Berden classification and only one patient was graded as IIIb in the ISKDC classification, the relatively mild kidney biopsy findings in the IgAV group might have contributed to the negative results. Nonetheless, the SS level in the IgAV group was observed to be lower than that in the non-SVKI disease group. IgAV patients with accompanying abdominal pain, indicating active vasculitis in the gastric tract, also exhibited a lower SS level than did those without the symptom. These results suggested that the SS level was associated with SVKI activity in patients with IgAV. However, our cohort study only included IgAV patients admitted to our nephrology department, all of whom exhibiting such kidney-involvement findings as hematuria, proteinuria, and acute eGFR decline. A biased selection of IgAV patients may have affected the results. Studies including IgAV patients with no or mild kidney-involvement findings will help build consensus on the SS level in IgAV patients.

Consistent with the results of our pilot study (2), the SS level was significantly lower in crescentic class patients in the AAV group. The detection ability of the SS level for such patients was sufficiently high, and the combined formulae of the SS level and other predictors showed higher detection results. Considering the limitations of other predictors, such as CRP level elevation by infection and the effort needed to evaluate BVAS, the SS level measurement may be useful for estimating kidney biopsy findings in AAV patients in some clinical situations.

In the GBM group, the SS level was unexpectedly similar among groups classified according to the Berden classification. Although the precise reason for this is unknown, insufficient statistical power might have influenced the results considering that only eight patients received a kidney biopsy. The SS level was by far the lowest in the GBM group, and the percentage of glomeruli with the cellular crescent finding was significantly correlated with the SS level, even with the limited statistical power. Therefore, a lower SS level may be associated with the severity of SVKI in patients with GBM. As this is the first study evaluating the SS level in GBM, further research is warranted on our preliminary findings.

In the *post-hoc* analysis on each sulfatide component, we witnessed that IgAN, AAV, and GBM patients had higher d18:2 and lower d18:1 proportions per total SS level than did donors. There are a variety of sulfatide types according to their sphingoid base structure, such as d18:1, d18:2, and t18:0, although the clinical function of each type remains unclarified. To the best of our knowledge, no study has assessed the sulfatide composition ratio in patients with vasculitis-related diseases. However, our results imply that the sulfatide component ratio can be associated with disease activity in SVKI. Furthermore, the proportions of d18:1 and d18:2 in this investigation were strongly correlated with crescentic findings in IgAN patients rather than in SVKI diseases. These results suggest that the sulfatide component ratio, rather than the total SS level, is more strongly associated with disease activity in some diseases. Clinical studies focusing on each sulfatide component are needed.

There were several limitations to this research. First, it was a retrospective study, and we cannot deny the possibility that unmeasured confounders influenced the results. Moreover, the information on physical and pathological findings was collected from medical records, which had been completed by numerous medical doctors and renal pathologists without unified rules for this research. Bias and inaccuracy of the data could have affected our findings; as an example, the pathological finding of fibrinoid necrosis in the renal artery and BVAS, both strong indicators of active SVKI disease, were not significantly associated with the SS level. Although we assumed that insufficient statistical power due to a limited sample size and assessment method (i.e., qualitative assessment of the fibrinoid necrosis finding and possible overestimation of vasculitis activity by BVAS due to mistaking chronic or burnt-out findings as active vasculitis) might have contributed to such results. Additional research is required to confirm the association between the SVKI disease activity and the SS level. Second, we assessed the association between the SS level and kidney biopsy findings only in patients who had received kidney biopsy. Therefore, it is uncertain whether our results on the association of the SS level with kidney biopsy findings can be applied in patients who do not undergo the procedure. Third, we could not detect an association between the SS level and the outcome endpoints of death or renal death. Although various confounders, such as differences in treatment strategy, presumably affected the results, it was unclear if the SS level measurement was useful for predictions of outcome development. Lastly, this research only included patients who were admitted to the nephrology department of a university hospital in Japan; the external validity of our results is limited. Prospective studies of patients from multiple centers and countries are desired.

In conclusion, the SS level appears to become decreased in more severe SVKI diseases. A lower SS level may also predict active crescentic lesions on kidney biopsy in patients with SVKI. The clinical potential of the SS level measurement warrants further investigation.

## Data availability statement

The original contributions presented in the study are included in the article/**Supplementary Material**. Further inquiries can be directed to the corresponding authors.

## Ethics statement

The studies involving humans were approved by Ethics committee of Shinshu University School of Medicine. The studies were conducted in accordance with the local legislation and institutional requirements. The requirement for written informed consent was waived because of the retrospective nature of the study.

## Author contributions

DA: Conceptualization, Data curation, Formal Analysis, Funding acquisition, Investigation, Methodology, Project administration, Software, Writing – original draft. MH: Conceptualization, Supervision, Writing – review & editing, Methodology, Data curation, Investigation. TNa: Writing – review & editing, Investigation, Conceptualization, Funding acquisition, Supervision. TNi: Methodology, Writing – review & editing, Data curation, Investigation, Formal Analysis, Software. KY: Data curation, Investigation, Writing – review & editing. YY: Data curation, Investigation, Writing – review & editing, Formal Analysis, Methodology, Software. KH: Supervision, Writing – review & editing. NT: Supervision, Writing – review & editing. YK: Writing – review & editing, Conceptualization, Funding acquisition, Supervision, Methodology.
