# Supplementary material for: Serum sulfatide level is associated with severe systemic vasculitis with kidney involvement
Source: Front Immunol. 2023 Dec 4;14:1271741. doi: 10.3389/fimmu.2023.1271741 (PMC10726124; doi:10.3389/fimmu.2023.1271741)
Supplement: Supplementary file 1 [file DataSheet_1.docx]

Supplementary Material

# Supplementary Figures

#
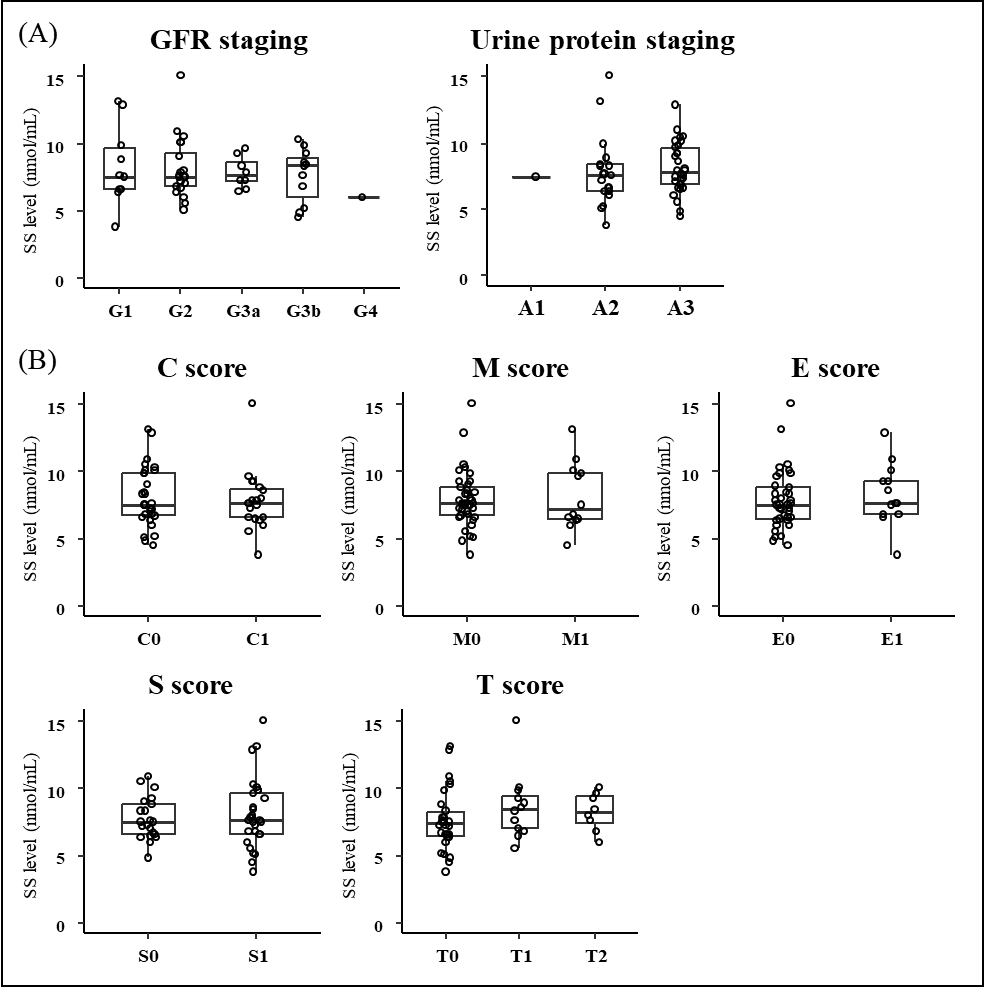


**Supplementary Figure S1.** SS level of the IgAN group according to (A) staging for GFR or urine protein level and (B) Oxford classification.


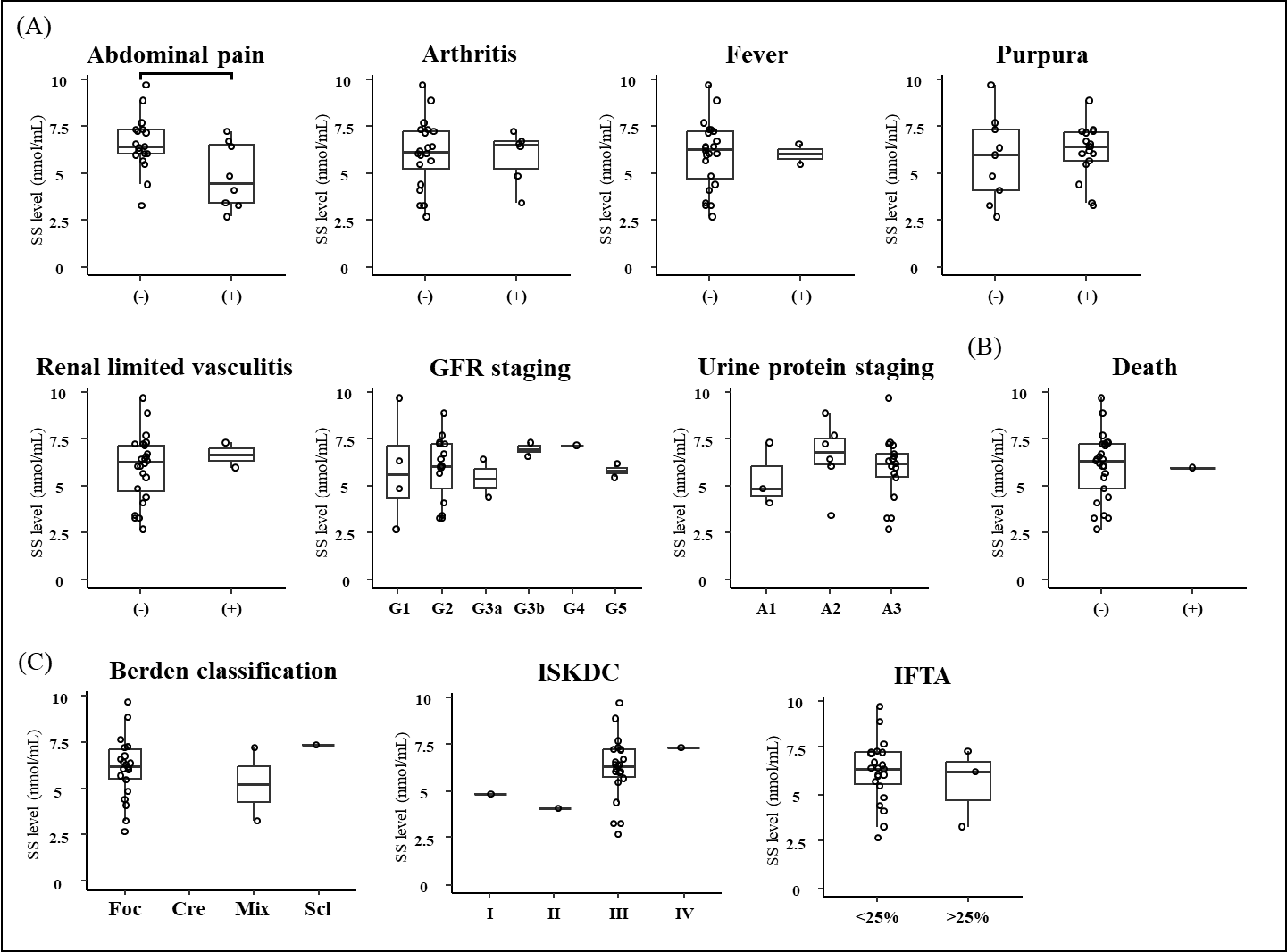


**Supplementary Figure S2.** SS level of the IgAV group according to (A) clinical findings, (B) outcome development, and (C) pathological findings on kidney biopsy.


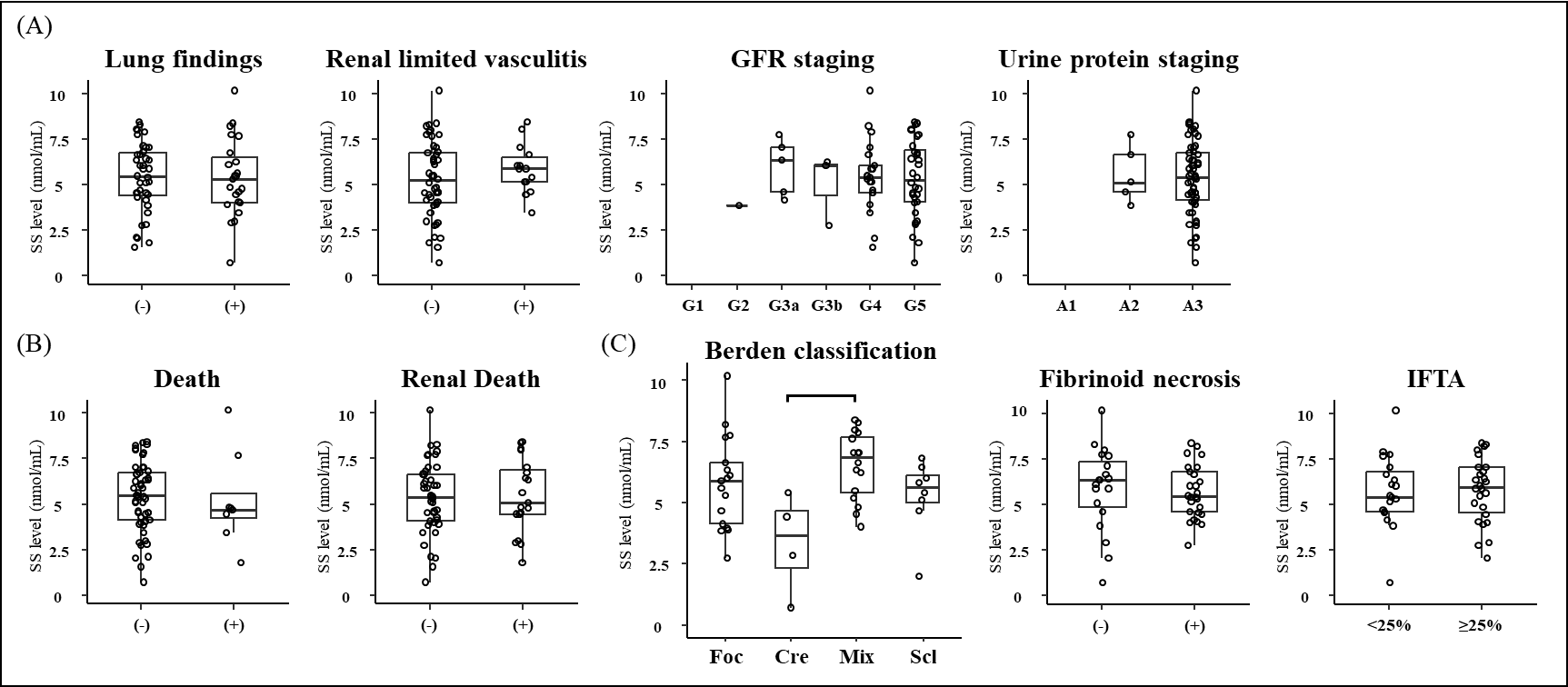


**Supplementary Figure S3.** SS level of the AAV group according to (A) clinical findings, (B) outcome development, and (C) pathological findings on kidney biopsy.


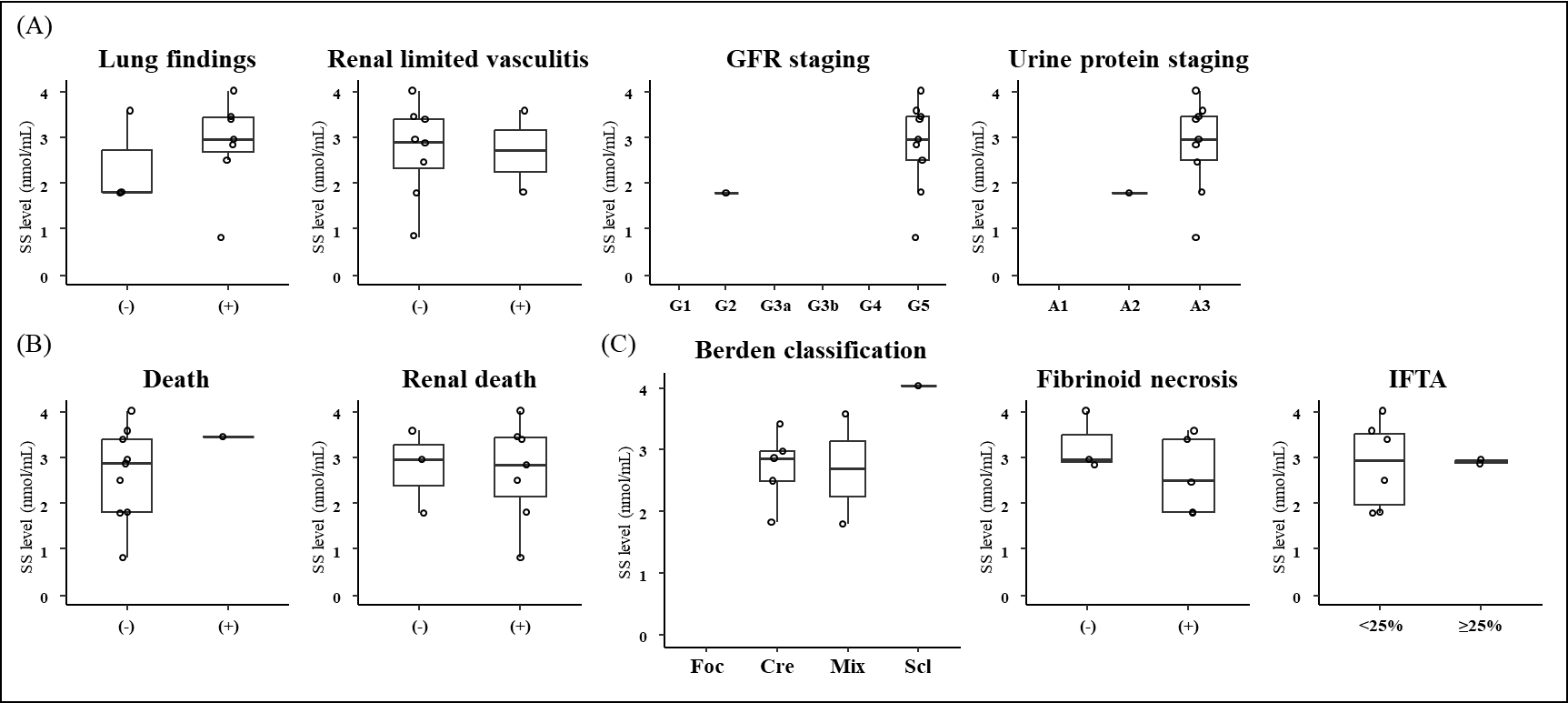


**Supplementary Figure S4.** SS level of the GBM group according to (A) clinical findings, (B) outcome development, and (C) pathological findings on kidney biopsy.


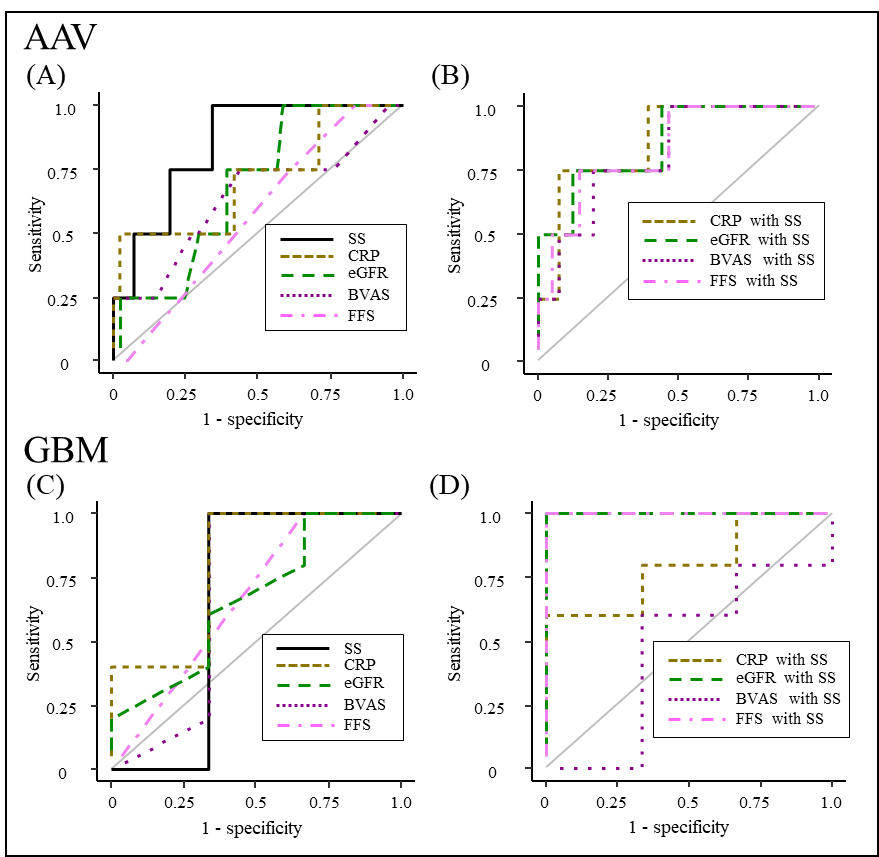


**Supplementary Figure S5.** ROC curves for SS level and other predictors in patients with the crescentic class kidney biopsy finding in Berden classification in the (A) AAV and (C) GBM groups. ROC curves for the combination of SS level and each predictor in the AAV and GBM groups are presented in (B) and (D), respectively.


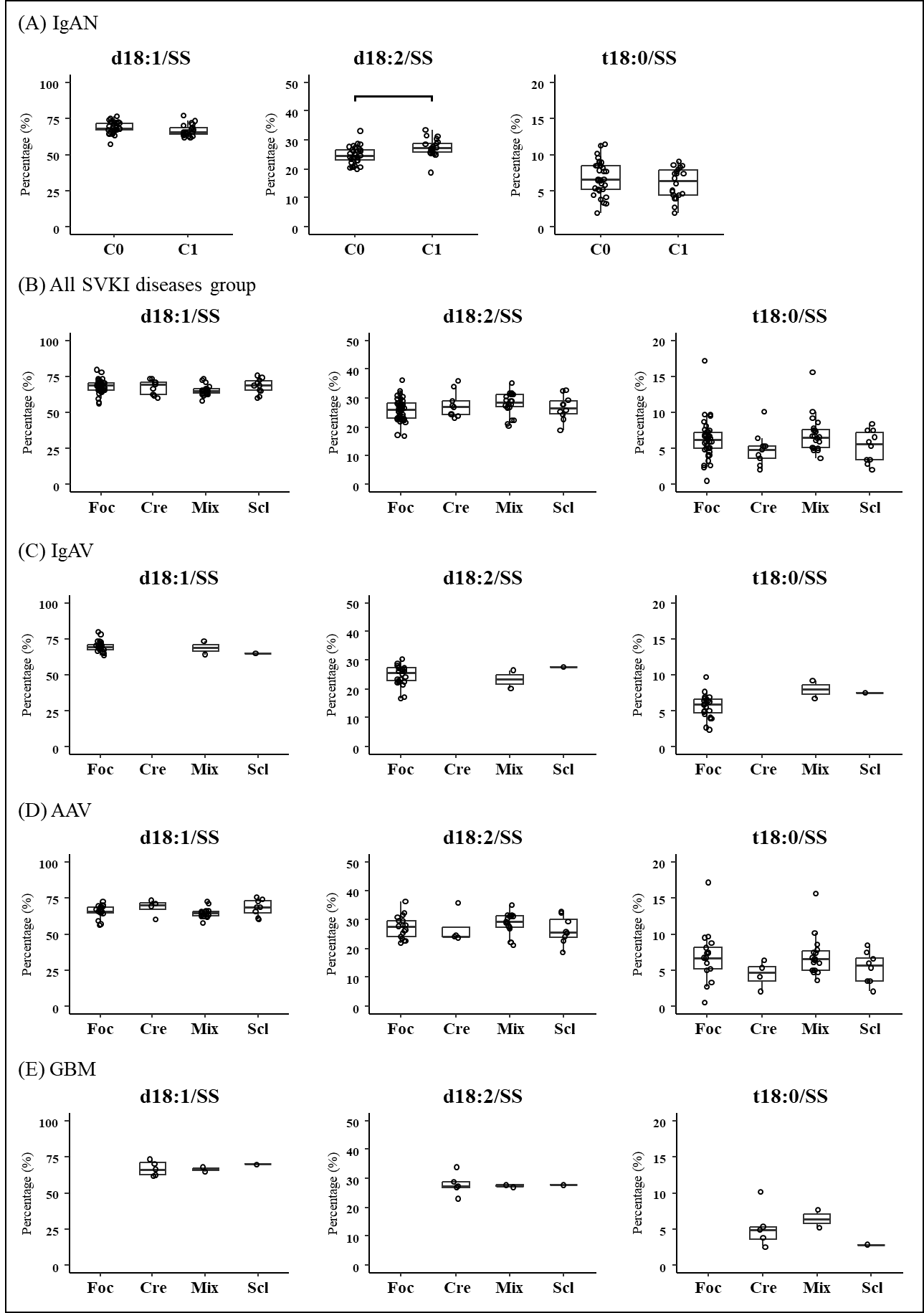


**Supplementary Figure S6.** Associations of sulfatide composition ratio with crescentic findings on kidney biopsy.

# Supplementary Tables

|  | Adjusted B of disease group for SS level | *p* |
| --- | --- | --- |
| IgAN to donors | -0.04 (-1.36, 1.28) | 0.95 |
| IgAV to donors | -2.25 (-3.24, -1.26) | <0.001 |
| IgAV to IgAN | -1.81 (-2.88, -0.75) | 0.001 |
| AAV to donors | -2.57 (-3.74, -1.39) | <0.001 |
| AAV to IgAN | -2.06 (-3.51, -0.62) | 0.006 |
| AAV to IgAV | -0.04 (-1.13, 1.04) | 0.94 |
| GBM to donors | -6.03 (-7.23, -4.82) | <0.001 |
| GBM to IgAN | -5.45 (-7.33, -3.56) | <0.001 |
| GBM to IgAV | -3.17 (-4.48, -1.85) | <0.001 |
| GBM to AAV | -2.78 (-4.15, -1.40) | <0.001 |

**Supplementary Table S1.** Partial regression coefficient with 95%CI of disease group for SS level after adjusting for age and sex.

|  | Adjusted B of SS level for crescentic findings in the SVKI diseases group with imputed dataset | *p* |
| --- | --- | --- |
| All-type crescents (%) | -0.03 (-0.05, 0.00) | 0.06 |
| Cellular or fibrocellular crescents (%) | -0.03 (-0.06, -0.01) | 0.01 |
| Cellular crescents (%) | -0.03 (-0.05, -0.01) | 0.002 |

**Supplementary Table S2.** Partial regression coefficient with 95%CI of SS level for parameters related to crescentic findings on kidney biopsy in the SVKI diseases group after adjusting for age, sex, eGFR, and serum ALT and TC levels.

|  | Donor | *p* | IgAN | *p* | IgAV | *p* | AAV | *p* | GBM | *p* |
| --- | --- | --- | --- | --- | --- | --- | --- | --- | --- | --- |
| Age (years) | 0.07 (-0.35, 0.47) | 0.75 | -0.01 (-0.29, 0.27) | 0.95 | -0.31 (-0.62, 0.09) | 0.13 | -0.22 (-0.44, 0.03) | 0.09 | 0.32 (-0.39, 0.79) | 0.37 |
| BMI (kg/m^2^) | -0.08 (-0.48, 0.34) | 0.71 | 0.07 (-0.21, 0.34) | 0.63 | -0.38 (-0.67, 0.01) | 0.06 | -0.07 (-0.32, 0.18) | 0.57 | 0.36 (-0.35, 0.81) | 0.31 |
| BVAS |  |  |  |  | 0.02 (-0.37, 0.41) | 0.92 | -0.12 (-0.36, 0.13) | 0.34 | -0.41 (-0.83, 0.30) | 0.24 |
| Laboratory data |  |  |  |  |  |  |  |  |  |  |
| Albumin (g/dL) | 0.40 (-0.01, 0.70) | 0.06 | -0.28 (-0.52, 0.00) | 0.05 | 0.36 (-0.04, 0.65) | 0.07 | 0.46 (0.24, 0.64) | <0.001 | 0.52 (-0.17, 0.86) | 0.13 |
| T-bil (mg/dL) | 0.19 (-0.41, 0.67) | 0.54 | -0.27 (-0.51, 0.01) | 0.06 | -0.11 (-0.48, 0.30) | 0.62 | 0.02 (-0.25, 0.29) | 0.88 | -0.56 (-0.88, 0.11) | 0.09 |
| AST (IU/L) | -0.19 (-0.56, 0.24) | 0.39 | -0.12 (-0.38, 0.17) | 0.42 | 0.02 (-0.37, 0.41) | 0.91 | -0.25 (-0.47, 0) | 0.05 | -0.47 (-0.85, 0.22) | 0.17 |
| ALT (IU/L) | -0.35 (-0.67, 0.07) | 0.10 | -0.28 (-0.53, -0.02) | 0.04 | -0.16 (-0.51, 0.24) | 0.44 | -0.18 (-0.41, 0.07) | 0.16 | -0.42 (-0.83, 0.29) | 0.23 |
| γ-GTP (IU/L) | -0.41 (-0.70, 0.00) | 0.05 | -0.41 (-0.62, -0.15) | 0.003 | -0.33 (-0.64, 0.07) | 0.10 | -0.22 (-0.44, 0.03) | 0.09 | -0.27 (-0.77, 0.44) | 0.45 |
| TC (mg/dL) | 0.39 (-0.04, 0.70) | 0.07 | 0.35 (0.08, 0.57) | 0.01 | 0.34 (-0.07, 0.65) | 0.10 | 0.67 (0.51, 0.79) | <0.001 | 0.78 (0.25, 0.95) | 0.02 |
| HDL-C (mg/dL) | 0.49 (0.08, 0.75) | 0.02 | 0.03 (-0.29, 0.35) | 0.85 | 0.50 (0.11, 0.76) | 0.01 | 0.46 (0.23, 0.65) | <0.001 | 0.70 (0.08, 0.93) | 0.03 |
| LDL-C (mg/dL) | 0.10 (-0.37, 0.53) | 0.69 | 0.35 (0.07, 0.58) | 0.01 | 0.20 (-0.21, 0.55) | 0.34 | 0.55 (0.34, 0.71) | <0.001 | 0.60 (-0.11, 0.90) | 0.09 |
| TG (mg/dL) | 0.02 (-0.40, 0.44) | 0.92 | -0.10 (-0.37, 0.19) | 0.51 | -0.16 (-0.52, 0.25) | 0.45 | 0.04 (-0.21, 0.3) | 0.73 | 0.63 (-0.05, 0.91) | 0.08 |
| eGFR (mL/min/1.73m^2^) | -0.23 (-0.59, 0.20) | 0.29 | -0.01 (-0.29, 0.27) | 0.93 | -0.03 (-0.42, 0.36) | 0.87 | 0.05 (-0.21, 0.29) | 0.72 | -0.28 (-0.77, 0.43) | 0.44 |
| CRP (mg/dL) | -0.12 (-0.51, 0.31) | 0.58 | 0.06 (-0.22, 0.34) | 0.66 | -0.51 (-0.75, -0.15) | 0.01 | -0.71 (-0.82, -0.57) | <0.001 | -0.40 (-0.82, 0.30) | 0.25 |
| Platelet (×10^4^/μL) | 0.21 (-0.22, 0.57) | 0.33 | 0.09 (-0.19, 0.36) | 0.53 | 0.23 (-0.17, 0.57) | 0.25 | -0.11 (-0.35, 0.14) | 0.40 | 0.12 (-0.55, 0.70) | 0.75 |
| D-dimer (μg/mL) | 0.29 (-0.31, 0.72) | 0.34 | 0.23 (-0.10, 0.52) | 0.16 | -0.47 (-0.76, -0.02) | 0.04 | -0.38 (-0.58, -0.13) | 0.00 | 0.30 (-0.41, 0.78) | 0.40 |
| Urine protein (g/gCr) | -0.06 (-0.46, 0.36) | 0.79 | 0.11 (-0.17, 0.38) | 0.43 | -0.04 (-0.42, 0.35) | 0.85 | 0.11 (-0.15, 0.35) | 0.40 | -0.03 (-0.65, 0.61) | 0.95 |
| Pathological findings |  |  |  |  |  |  |  |  |  |  |
| All-type crescents (%) | N/A | N/A | 0.14 (-0.15, 0.40) | 0.34 | 0.00 (-0.39, 0.40) | 0.99 | 0.08 (-0.22, 0.36) | 0.61 | -0.37 (-0.85, 0.46) | 0.37 |
| Cellular crescents (%) | N/A | N/A | 0.02 (-0.26, 0.30) | 0.89 | -0.08 (-0.46, 0.32) | 0.70 | -0.12 (-0.40, 0.18) | 0.43 | -0.75 (-0.95, -0.10) | 0.03 |
| Cellular or fibrocellular crescents (%) | N/A | N/A | 0.10 (-0.18, 0.37) | 0.49 | -0.14 (-0.51, 0.27) | 0.49 | -0.04 (-0.33, 0.25) | 0.78 | -0.11 (-0.76, 0.65) | 0.80 |
| Global sclerosis (%) | N/A | N/A | 0.28 (0.00, 0.52) | 0.05 | -0.22 (-0.57, 0.19) | 0.29 | 0.13 (-0.17, 0.41) | 0.39 | 0.34 (-0.48, 0.84) | 0.41 |
| Normal glomeruli (%) | N/A | N/A | -0.24 (-0.49, 0.04) | 0.09 | 0.10 (-0.31, 0.47) | 0.65 | 0.04 (-0.25, 0.33) | 0.78 | -0.04 (-0.72, 0.68) | 0.93 |
| IFTA (%) | N/A | N/A | 0.05 (-0.23, 0.33) | 0.71 | 0.07 (-0.33, 0.45) | 0.74 | -0.03 (-0.32, 0.27) | 0.86 | 0.24 (-0.56, 0.81) | 0.57 |

**Supplementary Table S3.** Correlation coefficient with 95%CI of SS level with parameters related to laboratory data and kidney biopsy findings in each disease group.

|  | Non-SVKI diseases | *p* | SVKI diseases | *p* | Donor | *p* | IgAN | *p* | IgAV | *p* | AAV | *p* | GBM | *p* |
| --- | --- | --- | --- | --- | --- | --- | --- | --- | --- | --- | --- | --- | --- | --- |
| T-bil (mg/dL) | -0.11 (-0.35, 0.14) | 0.37 | 0.04 (-0.17, 0.25) | 0.70 | 0.07 (-0.35, 0.47) | 0.75 | -0.27 (-0.51, 0.01) | 0.06 | -0.14 (-0.50, 0.26) | 0.50 | 0.01 (-0.24, 0.26) | 0.91 | -0.56 (-0.88, 0.11) | 0.09 |
| TC (mg/dL) | 0.36 (0.14, 0.55) | 0.002 | 0.62 (0.48, 0.73) | <0.001 | 0.28 (-0.15, 0.62) | 0.20 | 0.38 (0.11, 0.60) | 0.006 | 0.40 (0.02, 0.68) | 0.04 | 0.64 (0.46, 0.76) | <0.001 | 0.84 (0.45, 0.96) | 0.004 |
| HDL-C (mg/dL) | 0.20 (-0.06, 0.43) | 0.13 | 0.53 (0.36, 0.66) | <0.001 | 0.36 (-0.06, 0.68) | 0.09 | -0.06 (-0.33, -0.22) | 0.67 | 0.52 (0.17, 0.76) | 0.006 | 0.42 (0.18, 0.60) | <0.001 | 0.74 (0.20, 0.93) | 0.02 |
| LDL-C (mg/dL) | 0.32 (0.09, 0.52) | 0.008 | 0.49 (0.32, 0.63) | <0.001 | 0.24 (-0.19, 0.59) | 0.27 | 0.39 (0.12, 0.60) | 0.005 | 0.30 (-0.09, 0.62) | 0.13 | 0.54 (0.34, 0.70) | <0.001 | 0.65 (0.04, 0.91) | 0.04 |
| TG (mg/dL) | -0.08 (-0.31, 0.16) | 0.51 | 0.02 (-0.18, 0.22) | 0.85 | 0.01 (-0.41, 0.42) | 0.97 | -0.07 (-0.34, 0.22) | 0.65 | -0.09 (-0.46, 0.31) | 0.66 | 0.03 (-0.22, 0.28) | 0.83 | 0.66 (0.06, 0.91) | 0.04 |
| D-dimer (μg/mL) | 0.24 (-0.04, 0.48) | 0.10 | -0.43 (-0.59, -0.24) | <0.001 | 0.08 (-0.34, 0.48) | 0.71 | 0.25 (-0.03, 0.49) | 0.08 | -0.25 (-0.58, 0.16) | 0.23 | -0.36 (-0.56, -0.12) | 0.004 | 0.30 (-0.40, 0.78) | 0.40 |

**Supplementary Table S4.** Correlation coefficient with 95%CI of SS level related to laboratory parameters using imputed dataset for missing values.

| (A) | AAV | *p* | GBM | *p* |
| --- | --- | --- | --- | --- |
| SS | 0.86 (0.80, 1.00) | Ref | 0.67 (0.01, 1.00) | Ref |
| CRP | 0.71 (0.37, 1.00) | 0.28 | 0.80 (0.38, 1.00) | 0.40 |
| eGFR | 0.69 (0.44, 0.94) | 0.07 | 0.67 (0.21, 1.00) | >0.99 |
| BVAS | 0.64 (0.28, 1.00) | 0.07 | 0.70 (0.11, 0.80) | 0.95 |
| FFS | 0.57 (0.36, 0.79) | <0.001 | 0.67 (0.34, 0.99) | >0.99 |
| (B) | AAV | *p* | GBM | *p* |
| CRP with SS | 0.85 (0.66, 1.00) | 0.29 | 0.80 (0.45, 1.00) | >0.99 |
| eGFR with SS | 0.87 (0.67, 1.00) | 0.02 | 1.00 (1.00, 1.00) | 0.16 |
| BVAS with SS | 0.85 (0.68, 1.00) | 0.06 | 0.47 (0.00, 1.00) | 0.61 |
| FFS with SS | 0.85 (0.63, 1.00) | <0.001 | 1.00 (1.00, 1.00) | 0.05 |

**Supplementary Table S5.** AUC values with 95%CI of SS level for the crescentic class kidney biopsy finding in Berden classification in the AAV and GBM groups.

| (A) IgAN | d18:1/SS | *p* | d18:2/SS | *p* | t18:0/SS | *p* |
| --- | --- | --- | --- | --- | --- | --- |
| All-type crescents (%) | -0.29 (-0.52, -0.01) | 0.04 | 0.39 (0.13, 0.61) | 0.005 | -0.14 (-0.40, 0.14) | 0.33 |
| Cellular crescents (%) | -0.36 (-0.58, -0.09) | 0.01 | 0.43 (0.17, 0.63) | 0.002 | -0.06 (-0.33, 0.22) | 0.67 |
| Cellular or fibrocellular crescents (%) | -0.29 (-0.52, -0.01) | 0.04 | 0.39 (0.13, 0.60) | 0.005 | -0.16 (-0.42, 0.13) | 0.27 |
| (B) All SVKI diseases group | d18:1/SS | *p* | d18:2/SS | *p* | t18:0/SS | *p* |
| All-type crescents (%) | -0.23 (-0.43, -0.01) | 0.04 | 0.28 (0.06, 0.47) | 0.01 | -0.02 (-0.25, 0.20) | 0.84 |
| Cellular crescents (%) | -0.09 (-0.31, 0.14) | 0.44 | 0.15 (-0.07, 0.36) | 0.18 | -0.02 (-0.24, 0.21) | 0.88 |
| Cellular or fibrocellular crescents (%) | -0.20 (-0.40, 0.03) | 0.09 | 0.22 (-0.00, 0.42) | 0.05 | -0.02 (-0.24, 0.20) | 0.86 |
| (C) IgAV | d18:1/SS | *p* | d18:2/SS | *p* | t18:0/SS | *p* |
| All-type crescents (%) | -0.23 (-0.57, 0.18) | 0.27 | 0.10 (-0.31, 0.47) | 0.65 | 0.16 (-0.25, 0.52) | 0.45 |
| Cellular crescents (%) | -0.35 (-0.66, 0.05) | 0.08 | 0.23 (-0.18, 0.57) | 0.27 | 0.18 (-0.23, 0.54) | 0.39 |
| Cellular or fibrocellular crescents (%) | -0.25 (-0.58, 0.16) | 0.24 | 0.11 (-0.30, 0.48) | 0.60 | 0.17 (-0.25, 0.53) | 0.43 |
| (D) AAV | d18:1/SS | *p* | d18:2/SS | *p* | t18:0/SS | *p* |
| All-type crescents (%) | -0.06 (-0.35, 0.24) | 0.70 | 0.18 (-0.12, 0.45) | 0.24 | -0.15 (-0.43, 0.15) | 0.32 |
| Cellular crescents (%) | -0.10 (-0.20, 0.38) | 0.52 | 0.06 (-0.24, 0.34) | 0.72 | -0.14 (-0.42, 0.16) | 0.34 |
| Cellular or fibrocellular crescents (%) | -0.01 (-0.30, 0.28) | 0.95 | 0.08 (-0.22, 0.36) | 0.61 | -0.13 (-0.41, 0.17) | 0.40 |
| (E) GBM | d18:1/SS | *p* | d18:2/SS | *p* | t18:0/SS | *p* |
| All-type crescents (%) | -0.26 (-0.82, -0.54) | 0.53 | 0.19 (-0.59, 0.79) | 0.65 | 0.20 (-0.59, 0.79) | 0.63 |
| Cellular crescents (%) | 0.42 (-0.40, 0.87) | 0.30 | -0.63 (-0.92, 0.14) | 0.10 | 0.05 (-0.68, 0.73) | 0.91 |
| Cellular or fibrocellular crescents (%) | -0.37 (-0.85, 0.45) | 0.37 | 0.32 (-0.49, 0.84) | 0.43 | 0.01 (-0.70, 0.71) | 0.98 |

**Supplementary Table S6.** Correlation coefficient with 95%CI of sulfatide composition ratio with parameters related to crescentic findings on kidney biopsy.
